# Salivary protease spectrum biomarkers of oral cancer

**DOI:** 10.1038/s41368-018-0032-z

**Published:** 2019-01-03

**Authors:** Yun Feng, Qian Li, Jiao Chen, Ping Yi, Xin Xu, Yaping Fan, Bomiao Cui, Yu Yu, Xiaoying Li, Yue Du, Qianming Chen, Lingling Zhang, Jingjing Jiang, Xuedong Zhou, Ping Zhang

**Affiliations:** State Key Laboratory of Oral Diseases, National Clinical Research Center for Oral Diseases, West China Hospital of Stomatology, Sichuan University, Chengdu, China

## Abstract

Proteases are important molecules that are involved in many physiological and pathological processes of the human body, such as growth, apoptosis and metastasis cancer cells. They are potential targets in cancer diagnosis and biotherapy. In this study, we analyzed the salivary protease spectrum of patients with oral squamous cell carcinoma (OSCC), oral benign masses and chronic periodontitis, as well as that of health, using human protease array kits, enzyme-linked immunosorbent assay, western blot and immunofluorescence. The salivary protease spectrum was found to be associated with oral diseases. For example, the saliva of patients with OSCC contained increased numbers of proteases than those of other oral diseases and health. The levels of matrix metalloproteinase (MMP)-1, MMP-2, MMP-10, MMP-12, A disintegrin and metalloprotease (ADAM)9, A disintegrin and metalloprotease with thrombospondin type 13 motifs (ADAMST13), cathepsin V and kallikrein 5 in the saliva of patients with OSCC were significantly increased compared with those of other groups. Taking MMP-1, cathepsin V, kallikrein 5 and ADAM9 as biomarkers of OSCC, cutoff values were199, 11.34, 9.29 and 202.55 pg·mL^−1^, respectively. From the area under the curve, sensitivity and specificity, the combination of cathepsin V/kallikrein5/ADAM9 was an optimal biomarker for diagnosing OSCC. Thus, analysis of the salivary protease spectrum may be an innovative and cost-efficient approach to evaluating the health status of the oral cavity. Specifically, increases in cathepsin V, kallikrein 5 and ADAM9 may be useful biomarkers in the screening and diagnosis of OSCC.

## Cancer: Salivary enzymes show cancer signature

Patients with oral cancer have elevated levels of certain protease enzymes in their saliva, a finding that could help with screening and diagnosis. Xuedong Zhou and colleagues from Sichuan University, China, collected saliva samples from patients with different mouth diseases—a type of cancer known as oral squamous cell carcinoma, benign mouth lesions or mild chronic gum disease—as well as healthy controls. They tested the saliva for levels of 35 different proteases, a class of enzymes involved in cleaving proteins, and identified several proteases present only in the saliva of patients with oral cancer, three of which they showed were expressed at high levels in cultured oral tumor cell lines as well. These proteases could thus serve as useful biomarkers in a future spit test for early cancer detection and disease monitoring.

## Introduction

Laboratory testing is an important and accurate method of diagnosis and prognosis in the evaluation of human diseases. Some chronic diseases, such as cancer, may have progressed to intermediate or advantaged stages by the time of diagnosis, which results in a poorer prognosis; therefore, an earlier diagnosis is an important, although challenging, goal. Early diagnosis may be facilitated by developing accurate laboratory testing methods to support clinicians. Whole saliva is a fluid mixture comprised of water, organic and inorganic components secreted by the major and minor salivary glands, gingival crevicular fluid and serum, desquamated epithelial cells from the oral mucosa, as well as oral microorganisms and their products.^1–4^ It is a complex fluid containing a variety of hormones, antibodies, microorganisms, proteins, enzymes, and cytokines.^4^ Many advantages exist to utilizing saliva as a body fluid in laboratory tests as compared to serum and tissue samples: saliva not only contains a wide spectrum of biomarkers for various diseases, but the collection of saliva is noninvasive, its transport and storage are easy, and obtaining saliva is cost effective and efficient.^5–7^

With the development of salivaomics, increased numbers of biomarkers that are related to oral and systemic diseases have been identified in the saliva.^8–10^ Salivary diagnostics, as an effective modality for early screening, diagnosis, prognosis evaluation, and monitoring of therapy for oral and systemic diseases, has long been an attractive diagnostic and screening option for clinical doctors and basic researchers.^5,11^

Proteases are important molecules, cleaving proteins into smaller peptides at either the N-terminal or C-terminal regions, and are involved in many physiological and pathological processes.^12–14^ At present, about 500–600 different proteases have been identified in humans.^14,15^ Human proteases can be divided into threonine, serine, cysteine, aspartic and metalloprotease groups depending on their mechanism of proteolysis.^14–17^ The abnormal activation of proteases can generate pathological changes in cells, tissues and organs. Previous researches have shown that many proteases are associated with the metastasis and translocation of human cancers.^18,19^ Inhibitors of these proteases can alleviate invasion and metastasis of cancer cells.^20^

Oral cancer has emerged as a global public health problem due to its increasing incidence and mortality rate;^21–23^ moreover, delays in cancer diagnoses result in a higher mortality rate.^24,25^ Hence, advances in new screening and early detection technologies have become the most effective strategies to reduce deaths due to this disease.^26^ Unlike other deeply invasive cancers, oral cancer is located in the oral cavity in direct contact with saliva; therefore, sampling saliva is likely to be the most effective way to identify several sensitive and specific biomarkers of this disease in patients.^11^ In this study, we analyzed the saliva protease spectrum of patients with oral cancer and compared it with other oral diseases. Our results indicated that the protease spectrum of oral cancer was markedly distinct from that of healthy controls, as well as patients with oral benign masses (OBM) and mild chronic periodontitis (CPD).Thus, the analysis of the salivary protease spectrum may be a useful approach to screen and diagnose oral cancer during the evaluation of the health status of the oral cavity.

## Results

### Saliva of patients with OSCC show more protease types

**Fig. 1:**
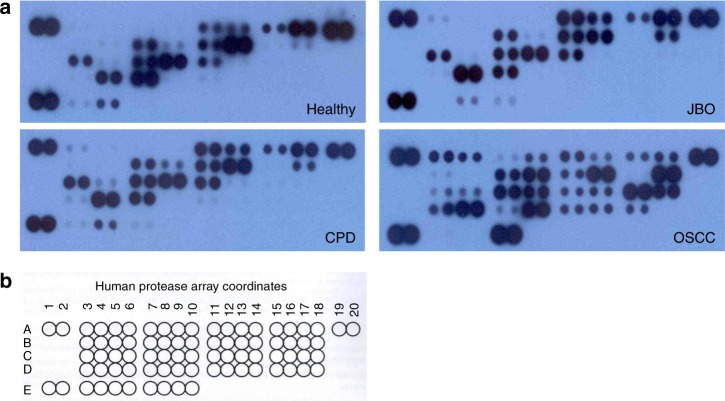
Analysis of salivary proteases by human protease array kits Stimulated saliva (0.5 mL) was collected in a salivate tube from healthy donors or patients with JBO, OSCC and CPD. Saliva proteases were detected using human protease array kits according to the manufacturer’s instructions. **a** Protease profile analyses using protease array kits. Pictures are representative of each of the four types of samples. **b** Each dot on the membrane represents a corresponding protease

The human protease array kit used in these studies measured 35 types of proteases (Fig. 1 and Table 1). We analyzed 16 saliva samples from healthy individuals, as well as patients with jaw bone ossification fibroma (JBO), OSCC and CPD using a protease array kit. As shown in Fig. 1, the salivary protease spectrum was quite different for the various oral conditions. More salivary proteases could be detected in patients with OSCC than in other diseases or the healthy group. We detected 30 proteases in patients with OSCC, 25 proteases in patients with CPD, 18 proteases in patients with JBO, and 17 proteases in healthy controls (Table 2). These results indicated that the salivary protease spectrum was highly related to the oral health status with the saliva of patients with OSCC having more proteases than that of patients with other oral diseases.

**Table 1:** Coordinates of proteases located on the human protease array membrane

	1/2	3/4	5/6	7/8	9/10	11/12	13/14	15/16	17/18	19/20
A	RS	ADAM8	ADAM9	ADAMTS1	ADAMTS13	Cathepsin A	Cathepsin B	Cathepsin C	Cathepsin D	RS
B		Cathepsin E	Cathepsin L	Cathepsin S	Cathepsin V	Cathepsin X/Z/P	DPPIV/ CD26	Kallikrein 3/PSA	Kallikrein 5	
C		Kallikrein 6	Kallikrein 7	Kallikrein 10	Kallikrein 11	Kallikrein 13	MMP-1	MMP-2	MMP-3	
D		MMP-7	MMP-8	MMP-9	MMP10	MMP-12	MMP-13	Neprilysin /CD10	Presenilin	
E	RS	Proprotein Convertase 9	Proteinase 3	uPA/ Urokinase	NC					

*RS* reference spot, *ADAM* a disintegrin and metalloprotease, *ADAMTS* a disintegrin and metalloproteinase with thrombospondin motifs, *MMP* matrix metalloproteinases, *uPA* urokinase-type plasminogen activator, *NC* negative control

**Table 2:** Proteases detected in saliva from patients with various oral diseases

Item	Healthy	JBO	CPD	OSCC
		ADAM8	ADAM8	ADAM8
			ADAM9	ADAM9
			ADAMTS1	ADAMTS1
				ADAMTS13
	Cathepsin A	Cathepsin A	Cathepsin A	Cathepsin A
	Cathepsin B	Cathepsin B	Cathepsin B	Cathepsin B
	Cathepsin C	Cathepsin C	Cathepsin C	Cathepsin C
	Cathepsin D	Cathepsin D	Cathepsin D	Cathepsin D
				Cathepsin E
Positive proteases	Cathepsin S	Cathepsin S	Cathepsin S	Cathepsin S
	Cathepsin V	Cathepsin V	Cathepsin V	Cathepsin V
	Cathepsin X/Z/P	Cathepsin X/Z/P	Cathepsin X/Z/P	Cathepsin X/Z/P
	DPPIV/CD26	DPPIV/CD26	DPPIV/CD26	DPPIV/CD26
	Kallikrein 5	Kallikrein 5	Kallikrein 5	Kallikrein 5
	Kallikrein 6	Kallikrein 6	Kallikrein 6	Kallikrein 6
			Kallikrein 7	Kallikrein 7
	Kallikrein 10	Kallikrein 10	Kallikrein 10	Kallikrein 10
	Kallikrein 11	Kallikrein 11	Kallikrein 11	Kallikrein 11
	Kallikrein 13	Kallikrein 13	Kallikrein 13	Kallikrein 13
				MMP-1
				MMP-2
				MMP-3
			MMP-7	MMP-7
	MMP-8	MMP-8	MMP-8	MMP-8
	MMP-9	MMP-9	MMP-9	MMP-9
				MMP10
			MMP-12	MMP-12
				MMP-13
			Neprilysin/CD10	Neprilysin/CD10
			uPA/Urokinase	uPA/Urokinase
	Proprotein convertase 9	Proprotein convertase 9	Proprotein convertase 9	
	Proteinase 3	Proteinase 3	Proteinase 3	
Total	17(48.6%)	18(51.4%)	25(71.4%)	30(85.7%)

*JBO* jaw bone ossification fibroma, *CPD* chronic periodontitis, *OSCC* oral squamous cell carcinoma, *ADAM* A disintegrin and metalloprotease, *ADAMTS* a disintegrin and metalloproteinase with thrombospondin motifs, *MMP* matrix metalloproteinases, *uPA* urokinase-type plasminogen activator

### Increased proteases in saliva of patients with OSCC

**Fig. 2:**
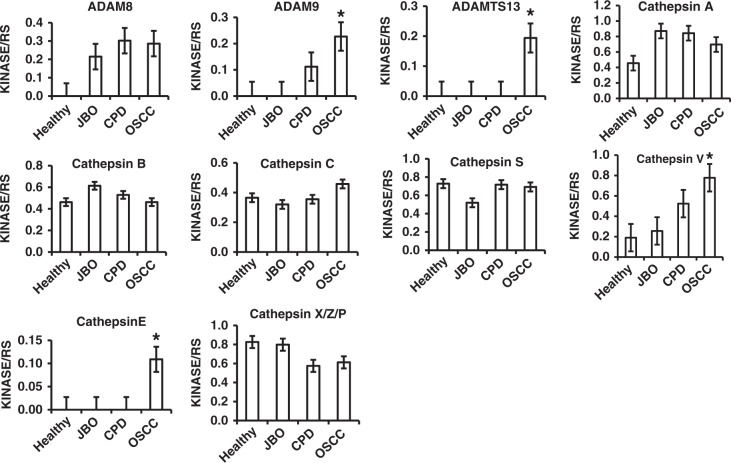
Quantitative analysis of ADAM and Cathepsin family by human protease array kits The protease profiles of 20 saliva samples from four healthy donors, and four patients each with JBO, OSCC and CPD were analyzed using a human protease array kit. The gray value of each dot on X-ray films, representing every protease in saliva, was measured via densitometry and Image J software. Numerical data are presented as the mean ± standard deviation (SD). The difference among the means was analyzed by one-way analysis of variance (ANOVA).*P < 0.05 compared with healthy donors, or patients with JBO or CPD

**Fig. 3:**
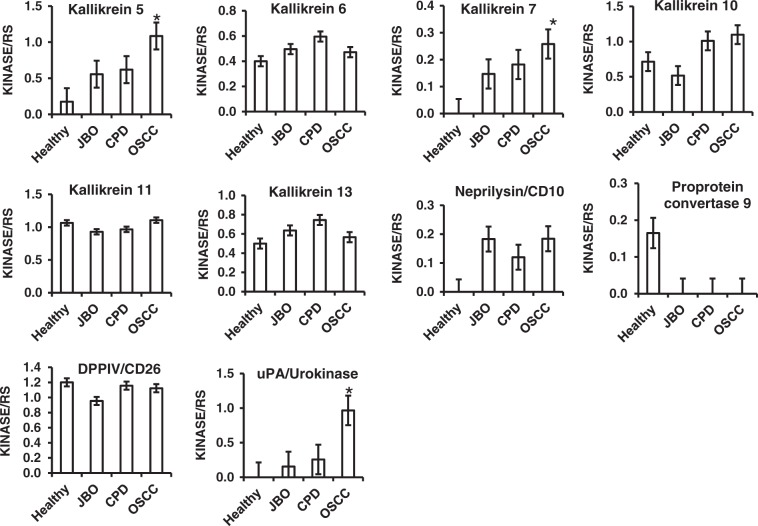
Quantitative analysis of kallikrein family, Neprilysin/CD10, Proprotein Convertase 9, DPPIV/CD26 and uPA by human protease array kits The protease profiles of 20 saliva samples from four healthy donors, and four patients each with JBO, OSCC and CPD were analyzed using a human protease array kit. The gray value of each dot on X-ray films, representing every protease in saliva, was measured via densitometry and Image J software. Numerical data are presented as the mean ± standard deviation (SD). The difference among the means was analyzed by one-way analysis of variance (ANOVA). *P < 0.05 compared with healthy donors, or patients with JBO or CPD

**Fig. 4:**
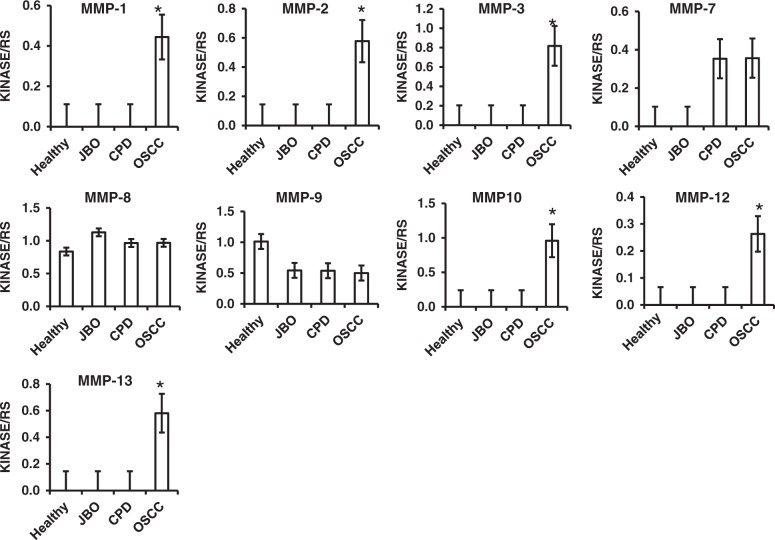
Quantitative analysis of MMP family by human protease array kits The protease profiles of 20 saliva samples from four healthy donors, and four patients each with JBO, OSCC and CPD were analyzed using a human protease array kit. The gray value of each dot on X-ray films, representing every protease in saliva, was measured via densitometry and Image J software. Numerical data are presented as the mean ± standard deviation (SD). The difference among the means was analyzed by one-way analysis of variance (ANOVA).*P < 0.05 compared with healthy donors, or patients with JBO or CPD

Our protease array kit was used to detect four members of the ADAM family, ten cathepsins, seven kallikreins, nine MMPs and five other protease families (Fig. 1 and Table 1). From the array kits, as shown in Fig. 1, and Tables 1 and 2, all four ADAM proteases were undetectable in the saliva of healthy donors, but were all found in the saliva of patients with OSCC. Among 35 kinds of proteases, ADAMST13, cathepsin E, and MMP-1, MMP-2, MMP-3, MMP-10, MMP-12 and MMP-13 were only detected in the saliva of patients with OSCC. Additonally, ADAM9, cathepsin v, kallikrein 5, urokinase plasminogen activator (uPA)/urokinase and kallikrein 7 were significantly increased in the saliva of patients with OSCC compared with that of healthy and in patients with CPD or JBO (*P* < 0.05 for all). In comparison, proprotein convertase 9 and proteinase 3 were detected in the saliva of patients with CPD or JBO and healthy controls, but not in the saliva of patients with OSCC (Fig. 1 and Figs. 2, 3, 4 Table 2).

### Kallikrein 5, Cathepsin V and ADAM9 as potential biomarkers in OSCC diagnosis

**Fig. 5:**
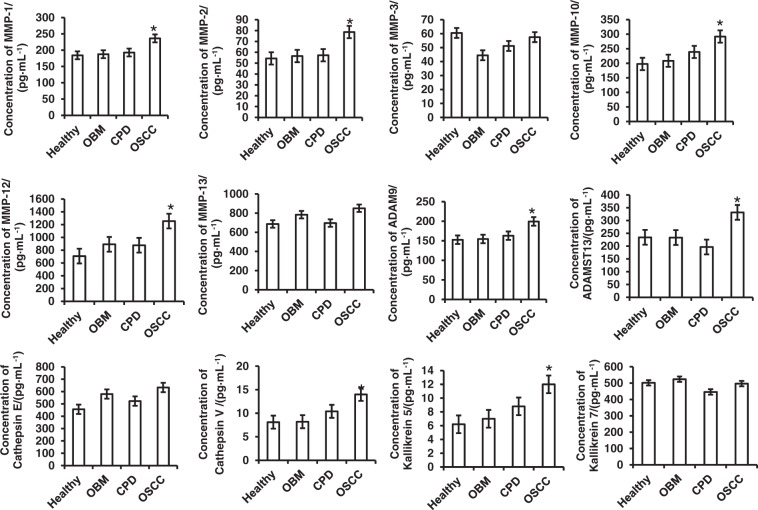
Quantitative analysis of salivary proteases by ELISA kits Eighty saliva samples from 20 each of healthy donors, and patients with OBM, OSCC and CPD were collected and the concentrations of MMP-1, MMP-2, MMP-3, MMP-10, MMP-12, MMP13, ADAM9, ADAMST13, cathepsin E, cathepsin V, kallikrein 5 and kallikrein 6 proteases were measured by ELISA kits. Numerical data are presented as the mean ± standard deviation (SD). The difference among the means was analyzed by one-way analysis of variance (ANOVA). *P < 0.05 compared with healthy donors, and patients with OBM or CPD

Based on the results of the human protease array kits, we used enzyme-linked immunoassay (ELISA) kits to analyse the concentrations of ADAMST13, cathepsin E, MMP-1, MMP-2, MMP-3, MMP-10, MMP-12 and MMP-13, which could only be detected in OSCC saliva, as well as ADAM9, cathepsin V, kallikrein 5 and kallikrein 7, which were significantly increased in OSCC saliva compared with those of healthy control, CPD and OBM. As shown in Fig. 5 and Table 3, all these proteases were detected by the ELISA kits. With regard to the results of the protease array kits, levels of MMP-1, MMP-2, MMP-10, MMP-12, cathepsin V, kallikrein 5,ADAM9 and ADAMST13 were also significantly increased compared with those of healthy or patients with CPD or OBM. However, MMP-3, MMP-7, MMP-9, MMP-13, cathepsin E and kallikrein 7did not show any significant differences between the four groups.

**Table 3:** Concentrations of saliva proteases detected by ELISA

Analyte/control	Healthy	JBO fibroma	OSCC	CPD
	(Mean OD)	(Mean OD)	(Mean OD)	(Mean OD)
ADAM8	0	0.101 ± 0.001	0.486 ± 0.110^a^	0.205 ± 0.03
ADAM9	0	0	0.367 ± 0.05^a^	0.11 ± 0.002
ADAMTS1	0	0	0.177 ± 0.009^a^	0.09 ± 0
ADAMTS13	0	0	0.294 ± 0.025^c^	0
MMP-1	0	0	0.304 ± 0.103	0
MMP-2	0	0	0.878 ± 0.315	0
MMP-3	0	0	0.718 ± 0.209	0
MMP-7	0	0	0.304 ± 0.115^a^	0.115 ± 0.091
MMP-8	0.835 ± 0.219	1.116 ± 0.306	0.978 ± 0.315	0.965 ± 0.298
MMP-9	1.012 ± 0.303	0.584 ± 0.198	0.156 ± 0.09^b^	0.538 ± 0.126
MMP-10	0	0	0.969 ± 0.312	0
MMP-12	0	0	0.365 ± 0.113^a^	0.12 ± 0.08
MMP-13	0	0	0.375 ± 0.105	0
Cathepsin A	0.456 ± 0.14	1.056 ± 0.320	0.697 ± 0.112	0.925 ± 0.325
Cathepsin B	0.462 ± 0.146	0.651 ± 0.151	0.262 ± 0.013^b^	0.768 ± 0.252
Cathepsin C	0.365 ± 0.231	0.35 ± 0.156	0.358 ± 0.178	0.325 ± 0.163
Cathepsin D	1.112 ± 0.352	1.125 ± 0.165	0.421 ± 0.154^b^	1.032 ± 0.257
Cathepsin E	0	0	0.103 ± 0.005^c^	0
Cathepsin L	0	0	0	0
Cathepsin S	0.728 ± 0.215	0.556 ± 0.124	0.592 ± 0.165	0.517 ± 0.201
Cathepsin V	0.19 ± 0.06	0.101 ± 0.004	0.878 ± 0.169^a^	0.05 ± 0.0
Cathepsin X/Z/P	0.628 ± 0.132	0.774 ± 0.268	0.312 ± 0.145^b^	0.575 ± 0.208
Kallikrein 3/PSA	0	0	0	0
Kallikrein 5	0.175 ± 0.112	0.272 ± 0.153	1.085 ± 0.312^a^	0.348 ± 0.114
Kallikrein 6	0.4 ± 0.105	0.472 ± 0.162	0.105 ± 0.05^b^	0.565 ± 0.151
Kallikrein 7	0	0	0.208 ± 0.101^a^	0.105 ± 0.04
Kallikrein 10	0.515 ± 0.231	0.547 ± 0.198	1.098 ± 0.305^a^	0.638 ± 0.246
Kallikrein 11	1.065 ± 0.289	0.985 ± 0.306	0.985 ± 0.268	0.745 ± 0.197
Kallikrein 13	0.5 ± 0.106	0.486 ± 0.113	0.266 ± 0.098^b^	0.698 ± 0.215
DPPIV/CD26	1.201 ± 0.316	0.857 ± 0.215	1.123 ± 0.298	1.157 ± 0.302
Neprilysin/CD10	0	0	0.394 ± 0.115	0.251 ± 0.108
Proprotein convertase 9	0.165 ± 0.05	0.201 ± 0.08	0	0.058 ± 0
Proteinase 3	0.415 ± 0.213	0.167 ± 0.09	0	0.383 ± 0.109
uPA/Urokinase	0	0	0.976 ± 0.296^d^	0.258 ± 0.107
Presenilin	0		0	0

*ADAM* a disintegrin and metalloprotease, *ADAMTS* a disintegrin and metalloproteinase with thrombospondin motifs, *MMP* matrix metalloproteinases, *uPA* urokinase-type plasminogen activator

^a,b^*P* < 0.05 compared with healthy controls or patients with CPD or OBM

^c^Only detected in OSCC saliva

^d^*P* < 0.05 compared with CPD

**Fig. 6:**
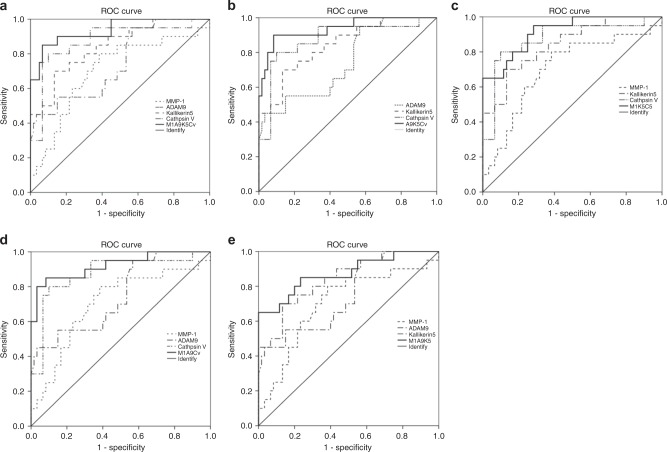
ROC curves of MMP-1, ADAM9, kallikrein 5, cathepsin V and their combination The saliva concentrations of MMP-1, ADAM9, kallikrein 5 and cathepsin V, which were detected by ELISA kit, were analyzed with ROC curves. **a** All of the four proteases and their combination; **b** ADAM9, kallikrein 5, cathepsin V and their combination (A9K5Cv); **c** MMP-1, kallikrein 5, cathepsinV and their combination (M1K5Cv); **d** MMP-1, ADAM9, cathepsin V and their combination (M1A9Cv); **e** MMP-1, ADAM9, kallikrein 5 and their combination (M1A9K5). Compared with the protease alone, all of the AUCs of combinations were significantly increased compared with those of proteases alone

Based on the ELISA results, MMP-1, MMP-2, MMP-10, MMP-12, cathepsin V, kallikrein 5 and ADAM9, which significantly increased in the saliva of patients with OSCC, were selected to calculate the cutoff point, Area Under Curve (AUC), sensitivity and specificity with Receiver Operating Characteristic (ROC). MMP-1, ADAM9, cathepsin V and kallikrein5, whose AUC was over 0.7, were selected as representative of different protease families and as potential biomarkers for the diagnosis of OSCC. As shown in Table 4 and Fig. 6, the cutoff points of MMP-1, ADAM9, cathepsin V and kallikrein 5 were 199, 202.55 and 11.34 and 9.29 pg·mL^−1^, respectively. Although the AUCs of each protease and their combination were over 0.7 (*P* < 0.05), the combination of MMP-1/ADAM9/cathepsinV/ kallikrein 5 (M1A9CvK5, Table 4 and Fig.6a), MMP-1/cathepsinV/ kallikrein5 (M1CvK5, Table 4, Fig.6b) and ADAM9/cathepsinV/kallikrein5 (A9CvK5, Table 4, Fig.6c) were more significant in the diagnosis of OSCC (all of their AUC were over 0.9). Combining the sensitivity and specificity (Table 4) of each protease and their combination, we found that A9CvK5, the combination of ADAM9/cathepsin V/kallikrein 5, is the optimal potential biomarker in diagnosing OSCC.

**Table 4:** AUC, cutoff point, sensitivity and specificity of MMP-1, ADAM9, cathepsin V and kallikrein 5 in the diagnosis of OSCC

Proteases	AUC	95%CI	*P*	Cutoff point	Sensitivity	Specificity
MMP-1	0.703	0.563--0.843	0.007	199	0.8	0.617
ADAM9	0.747	0.621--0.873	0.001	202.55	0.45	0.767
Cathepsin V	0.776	0.777--0.875	0	11.34	0.6	0.8
Kallikrein 5	0.843	0.743--0.942	0	9.29	0.7	0.867 7
M1A9CvK5	0.963	0.872--1.0	0		0.85	0.933
M1A9Cv	0.821	0.843--0.999	0		0.7	0.867
M1A9K5	0.873	0.773--0.974	0		0.65	1
M1CvK5	0.926	0.858--0.994	0		0.65	1
A9CvK5	0.938	0.873--1.0	0		0.9	0.991 7

*ADAM* a disintegrin and metalloprotease, *MMP* matrix metalloproteinases, *AUC* area under curve, *CI* confidence interval, *M1A9CvK5* MMP-1/ADAM9/Cathepsin v/Kallikrein 5, *M1A9Cv* MMP-1/ADAM9/Cathepsin v, *M1A9K5* MMP-1/ADAM9/Kallikerin5, *M1CvK5* MMP-1/Cathepsin v /Kallikrein 5, *A9CvK5* ADAM9/Cathepsin v/Kallikrein

### Kallikrein5, Cathepsin V and ADAM9 are expressed at high levels in oral tumor cell lines

**Fig. 7:**
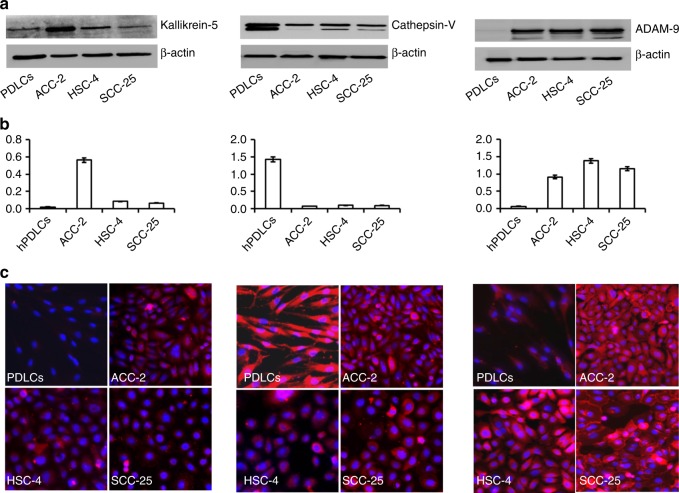
High expression of ADAM9, cathepsin V and kallikrein 5 in oral cancer cell lines Cancer cell lines were cultured in RPMI1640 or DMEM/F12 with 10% fetal bovine serum (FBS). Total protein was collected from cell lines and the expression of ADAM9, cathepsin V or kallikrein 5 was detected via western blot. Intracellular protein expression was analyzed by immunofluorescence. **a** The expression of kallikrein 5, cathepsin V or ADAM9 protein in cancer cell lines was analyzed by western blot. β-actin was used as a loading control. **b** Quantitative analysis of western blots. **c** Intracellular expression (red) of kallikrein 5, cathepsin V or ADAM9 in cancer cells lines as analyzed by immunofluorescence. Blue (nuclear) DAPI stain. Images are representative of three independent experiments. Numerical data are presented as the mean ± standard deviation (SD). *HSC-4 and SCC-25* oral squamous carcinoma cell lines, *ACC-2* oral adenocarcinoma cell line, *PDLCs* non-cancer cell line. *P *<* 0.05 compared with PDLCs. ^#^*P* < 0.05 compared with PDLCs

We sought to confirm that salivary proteases originated from cancer cells. We, therefore, analyzed the expression of kallikrein 5, cathepsin Vand ADAM9 in oral squamous cell carcinoma cell lines, HSC-4 and SCC-25, as well as the oral adenocarcinoma cell lineACC-2, by western blot and immunofluorescence. Human PDLC cells were used as a non-cancer cell control. As shown in Fig. 7, kallikrein 5, cathepsin V and ADAM9were expressed at a higher level in all tumor cell lines. Western blot and immunofluorescence results show that PDLCs expressed a high level of cathepsin V also. However, although kallikrein 5 could be detected in PDLCs by western blot, it was not detected by immunofluorescence.

## Discussion

Proteases are important molecules that cleave proteins into smaller peptides at either the N-terminal or C-terminal end. They typically function within the cell or, via secretion, play important roles in the extracellular environment.^27^ Human proteases are subdivided into five categories on the basis of their structure and proteolytic mechanism: serine proteases, cysteine proteases, aspartate proteases, metalloproteinases and threonine proteases. Protease signaling pathways are strictly controlled and the abnormal activation or secretion/release of proteases may give rise to pathological processes in cells, tissues and organs. Many proteases play important roles in the invasion and metastasis of cancers because of their ability to degrade the extracellular matrix barrier surrounding tumors.^15,16^ Proteases in bodily fluids or within cells may be useful as biomarkers in the screening, early diagnosis, and monitoring of cancer occurrence and progression. The human protease array kit was able to detect 35 types of proteases in human bodily fluids, including four members of the ADAM family proteases, nine types of cathepsin proteases, seven kallikrein protease family members, nine MMP protease family members, as well as protease 3 and proprotein convertase 9 (Fig. 1 and Table 1).

Saliva is comprised of a number of complex components which originate from the salivary glands and, as well as other compounds that may come from the oropharynx, gastrointestinal reflux, gingival crevicular fluid and blood.^2–4^ For many years, it has been recognized that analysis of the components of saliva can be a very effective tool for monitoring health status.^28^ Variation in saliva compounds has become the “window” that reflects the body’s physiological and pathological statuses. Therefore, the analysis of compounds within saliva may be useful for the monitoring of the onset of both oral and systemic disease, progression, recurrence, and treatment. With the advance of salivaomics, increased numbers of biomarkers of oral and systemic diseases are being identified in the saliva.^29,30^ Currently, it is commonly accepted that saliva is a useful body fluid that can be used for clinical and laboratory diagnoses, not only because it comprises a lot of disease biomarkers, but also because saliva is abundant and can easily be collected through noninvasive and painless procedures.^10^

OSCC accounts for over 95% of oral cancers and is the sixth greatest global public health problem due to its increasing incidence and mortality rate world wide.^31,32^ Unlike other more invasive cancers, oral cancers are located in the oral cavity, which facilitates the clinician’s access to the lesion for biopsy and a definitive diagnosis of the neoplasia; it also has direct contact with saliva. Thus, molecules, including proteases that are secreted and released by cancer cells, enter directly into the saliva. This means that it may be easier to detect several sensitive and positive biomarkers for oral cancer in saliva rather than serum. Thus, salivary screening of the protease spectrum may be an auxiliary tool in diagnosis.

Our results (Fig. 1, Fig. 2, Fig. 3, Fig. 4 and Table 2) demonstrate that the protease profile in saliva is quite different depending on oral status. The protease profile and levels in the saliva of patients with OSCC were significantly higher than in the saliva of non-cancer patients; for example, we identified 30 types of proteases in saliva from patients with OSCC as compared to only 17 proteases in saliva from healthy donors, 18 in saliva from patients with JBO, and 25 in saliva of patients with mild CPD. Among the 35 types of protease that were detected by protease array kits, including ADAMST13 and cathepsin E, as well as others to a total of eight types of proteases were detected in OSCC saliva (Fig. 1, Table 2). Another five types of proteases were also detected in saliva of patients with other oral diseases or in healthy controls, but were significantly increased in patients with OSCC (Figs. 2, 3, 4). These data indicate that the protease profile in saliva was associated with a persons’ oral health status. We are hopeful that salivary protease spectrum analysis may become a useful method for oral cancer screening and early diagnosis.

We confirmed results detected by protease array kit using ELISA kits. We collected 80 saliva samples from 20 healthy controls, as well as patients with OSCC, mild CPD and oral benign masses, and analyzed those proteases that were detectable or significantly increased in OSCC saliva according to protease array kits. It is difference with the array kits, all of that protease can be detected by ELISA kits (Fig. 5 and Table 3). As shown with the array kits, MMP-1, MMP-2, MMP-10, MMP-12, cathepsin V, kallikrein 5, ADAM9 and ADAMST13 were significantly increased in OSCC saliva compared with that of healthy donors, and patients with CPD or oral benign masses using ELISA kits (Fig. 5 and Table 3). Although MMP-1, MMP-2, MMP-3, MMP-10, MMP-13, cathepsin E could not be detected by array kits in the saliva of healthy donors, and patients with CPD and oral benign masses, all of these could be detected by ELISA methods. In comparison, MMP-3, MMP-13, cathepsin E and kallikrein7 were significantly increased when detected by array kits, but a significant difference among them was not found when detected by ELISA kit (Figs. 2, 3, 4 and Fig. 5, Table 3). Because the sensitivity of the ELISA is greater than that of dot plots, the existence of inconsistencies between the results of them is unsurprising.

ADAM and MMP protease families are involved in the maintenance and remodeling of the structure of extracellular matrix.^33,34^ Both types of proteases participate in the degradation of the extracellular matrix to promote the invasion and metastasis of cancer cells.^35,36^ Cathepsin protease family members are primarily cysteine proteases. In humans, there are 11 cysteine cathepsin family members^37^ that cleave their substrates in a relatively unspecific manner. Meanwhile, because of their function in lysosomal protein degradation, cathepsins play significant roles in the pathological processes of variety diseases such as cancer, periodontitis, and inflammation.^38,39^ Kallikreins or tissue kallikrein-related peptidases are serine proteases of the chymotrypsin family. The first identified member of the kallikrein family was found to be the most common protease in the pancreas; however, in fact, kallikrein protease family members are expressed in many different human organs. They function in a broad spectrum of physiological processes such as blood pressure regulation, tissue remodeling, and inflammatory cascades.^40^ This indicates that this salivary protease can increase invarious physiological and pathophysiological situations. For this reason, we selected patients with mild CPD or oral benign masses, and healthy donors as controls. ELISA analyses indicated that salivary proteases increased not only in saliva of patients with OSCC, but also in patients with CPD or oral benign masses. In order to identify an appropriate biomarker for the differential diagnosis of OSCC, we used ROC curves to calculate the cutoff point, AUC, sensitivity and specificity of each protease. MMP-1, ADAM9, cathepsin V and kallikrein 5 were selected because the relevant AUC was over 0.7 (Fig. 6, Table 4).The cutoff points forMMP-1, ADAM9, cathepsin V and kallikrein 5 were 199, 202.55, 11.34 and 9.29 pg·mL^−1^, respectively (Table 4). From the AUC, sensitivity and specificity, we found that the combination of ADAM9, cathepsin V and kallikrein 5 was a potential biomarker for diagnosing OSCC.

Salivary proteases usually originate from oral mucosa tissue, glands or oral microorganisms. Western blot and immunofluorescence results indicated that ADAM9, cathepsin V and kallikrein 5 were expressed at high levels in oral cancer cell lines. It is noteworthy that cathepsin V was also expressed in PDLCs, non-cancer cell lines isolated from healthy teeth, at a high level (Fig. 7). Combined with the ELISA results, we think that this protease is normally expressed in oral tissue. The types of salivary proteases that increased during oral disease differed according to the type of oral disease. The use of individual proteases as biomarkers of oral disease was not feasible

Presently, because of the relationship between saliva and OSCC, exploring the potential biomarker in saliva of patients with OSCC attract a lot of researchers and dentists. In the last decade, many potential biomarkers, such as mRNA, metabolites and cytokines that relate to disease, have been found in the saliva of patients with OSCC.^9,11,19^ Unfortunately, because of sensitivity and specificity, as well as technical requirements and cost, the use of such potential biomarkers has been confined to the laboratory. Future investigations will continue along different pathways using different techniques. In this study, we have targeted proteases involved in many cellular physiological and pathological processes. Our investigations have identified salivary proteases as potential biomarkers of OSCC; at the same time, we have provided a new way of obtaining biomarkers of OSCC with reasonable sensitivity and specificity.

## Methods and materials

### Saliva collection

Sixteen patients were enrolled in our protease array analysis, including those with JBO (*n* = 4), OSCC (*n* = 4), and CPD (*n* = 4), as well healthy controls (*n* = 4). Another eighty patients were enrolled in the ELISA quantitative assessment, including healthy controls (*n* = 20), and patients with OSCC (*n* = 20), mild CPD (*n* = 20) and OBM (*n* = 20); including: four parotid adenolymphomas, two myoepithelioma parotids, four keratocystic odontogenic tumors, three palatal pleomorphic adenomas, one mandible ameloblastic fibroma, four JBO, one basal cell adenoma parotid and one mandible epulis). All patients with OSCC or OBM were selected by pathological diagnosis. All patients with OSCC were T0N0M0, and their saliva was collected before surgery. All volunteers were informed of the aims and procedures of this study and that their medical data were to be used for our research only. All volunteers were also informed that the authors would visit them to identify individual medical information, during or after data collection. Patients with systemic or salivary gland diseases, or those currently taking non-steroidal anti-inflammatory drugs, antibiotics, or similar hormonal compounds, smokers, those who had less than 26 teeth or had received scaling and root planning in the previous 6 months were excluded.

Each participant routinely underwent thorough hepatic, renal, and hematologic clinical biochemical examinations. Stimulated saliva was collected with salivette tubes (Sarstedt AG & Co., Numbrecht, Germany). Before sample collection, participants were required to gargle. After 30 min, they were instructed to put the cotton from the salivette tube into their mouths for 10 min, without chewing the cotton, and then to spit the cotton back into the tubes. The tubes were kept on ice and sent to the laboratory immediately. Samples were centrifuged at 10,000×*g* for 10 min at 4 °C and the supernatants were stored at −80 °C until use. All samples were collected between 8:30–11:30 a.m. from Jan 1 2015 to Dec 30 2016.

### Protease array and image analysis

In this study, 0.5 mL saliva from each volunteer was tested using a Human Protease Array Kit (R&D Systems, Inc Minneapolis, USA), in accordance with the manufacturer’s instructions. For image analysis, X-ray film (Carestream health Inc. Wuhan, China) was scanned with a CanoScan LiDE 700 F scanner (Canon, Beijing, China) and analyzed by Image J (National Institutes of Health, Bethesda, Maryland, USA). The analysis of each sample was repeated using two array membranes.

### Quantitative assessment of salivary protease by ELISA

Assessment of protease levels in saliva were performed using human protease ELISA kits (Wuhan Colorful Gene Biological Technology Co, Ltd, China), in accordance with the manufacturer’s instructions. Briefly, the ELISA kits were removed from the refrigerator and the standards diluted to the desired concentration according to the manufacturer’s instructions. Then, 50 μL of diluted standards, as well as 50 μL of saliva samples were added into micro ELISA plates. Each sample included triplicate wells. The plates were then incubated at 37 °C for 30 min, follow by washing with wash buffer five times. Soon after, 50 μL HRP-conjugate reagent was added to each well, except the blank control well, and the plates incubated at 37 °C for another 30 min.Taking out the plates and washing five times, 50 μL of Chromogen Solution A and 50 μL Chromogen Solution B was added to each well, mixed by gently shaking and incubated at 37 °C for 15 min. Then, 50 μL stop solution was added to each well to terminate the reaction, the absorbance read at an OD of 450 nm using a Microtiter Plate Reader and the protease concentration calculated.

### Periodontal ligament cell isolation and culture

Periodontal ligament (PDL) tissues were obtained from premolar teeth and extracted for orthodontic purposes from three donors at the West China Hospital of Stomatology of Sichuan University. Protocols regarding the use and manipulation of PDL tissues were approved by the Institutional Review Board of West China Hospital of Stomatology, Sichuan University (WCHS-IRB-D-2015-147) and written informed consent was obtained from the donors. The extracted teeth were placed in and rinsed with Dulbecco’s Modified Eagle Medium (DMEM, Hyclone, USA) supplemented with 100 IU·mL^−1^ penicillin and 100 μg·mL^−1^ streptomycin. The remaining procedures were performed as described by Arnold et al.^41^ Briefly, PDLs attached to the middle third of the root were removed with a curette to avoid contamination with gingival and apical tissues. The PDL tissues were cut into ~1 mm^2^ pieces and placed in 25 mm^2^ culture flasks for cell culture in DMEM medium supplemented with 10% fetal bovine serum (FBS; Thermo Fisher Scientific Waltham, MA, USA), 100 U·mL^−1^ of penicillin and 100 μg·mL^−1^ of streptomycin at 37 °C, under 5% CO_2_ and 95% humidity. After reaching confluence of ~75% (in ~7 days), the cells were treated with 0.25% trypsin–0.1% EDTA (Hyclone; GE Healthcare Life Sciences, Marlborough, MA, USA) for cell passaging. PDLCs at the 5th or 6th passage were used for the present studies.

### Western blotting

HSC-4, ACC-2, and SCC-25 tumor cell lines were gifts from Professor Qian Ming, who purchased these from the ATCC and cultured them at the State Key Laboratory of Oral Diseases. All the cancer cell lines were cultured in medium with 10% FBS, 100 IU·mL^−1^ penicillin, and 100 μg·mL^−1^ streptomycin. The cells were maintained as monolayers in 25 cm^2^ plastic tissue culture flasks at 37 °C in a humidified atmosphere containing 5% CO_2_. Exponentially growing cells were used in all experiments. All cells were harvested and lysed in a buffer (50 mmol·L^−1^ Tris–HCl, pH 8.0; 5 mmol·L^−1^ EDTA; 150 mmol·L^−1^ NaCl; 0.5% Nonidet P-40; 0.5 mmol·L^−1^ PMSF; 0.5 mmol·L^−1^ DTT; and proteinase inhibitor mixture) for 30 min at 4 °C. Equal amounts of total protein (50 μg) were subjected to 12% SDS–polyacrylamide gel electrophoresis (PAGE) separation before transfer to Immun-Blot polyvinylidene fluoride membranes using a semi-dry transfer system (BioRad, Hercules, CA, USA). Membranes were blocked with 5% milk in Tris buffered saline (TBST; 150 mmol·L^−1^ NaCl; 50 mmol·L^−1^ Tris, pH7.4; 0.1% Tween-200) for 1 h at room temperature. The membranes were incubated with a 1:2 500 dilution of anti-ADAM9, anti-kallikrein 5, anti-cathepsin V antibody (abcam, Shanghai, China) and with a 1:1 000 dilution of anti–β-actin antibody (abcam) overnight, followed by washing with TBST. Bound antibodies were detected by incubation with corresponding horseradish peroxidase-conjugated secondary antibodies (ZSGB-Bio, Beijing, China) at a 1:2000 dilution for 120 min at room temperature. After extensive washing in TBST, the signal was detected by an Easy ECL western blot kit (TransGen Biotech, China). Band intensity was captured using a Chemidox XRS (BioRad).

### Immunofluorescence

HSC-4, ACC-2 and SCC-25 tumor cell lines were cultured on cover slips overnight. The cover slips were gently washed thrice in warm PBS. Cells were fixed with 4% formaldehyde for 15 min at room temperature, then permeabilized with 0.2% TritonX-100 (diluted in PBS) for 20 min and washed three times in PBS for 5 min each. The cells were then blocked with 1% BSA for 30 min at room temperature, and then incubated in rabbit anti-ADAM9, anti-kallikrein or anti-cathepsin V antibody (1:2500 diluted in 1% BSA) overnight at 4 °C. The coverslips were washed with PBS to remove unbound primary antibody, then incubated in Alexa Flour 555-phalloidin secondary antibody (Invitrogen, Carlsbad, CA, USA), diluted to 1:500 in 1% BSA, and incubated for 1 h at room temperature. Finally, after washing with PBS, the cover slips were mounted with Anti-Fade Fluorescence Mounting Medium with DAPI (HelixGen, Guangzhao, China) and photographed with a fluorescence microscope (Nikon Eclipse 80i; Nikon, Tokyo, Japan).

### Data availability

All of the data, material and methods which supporting the results can be found in the artic.

### Statistical analysis

SPSS 19.0 software (Chicago, IL, USA) was used for data analysis. Data was analyzed using a one-way analysis of variance (ANOVA; based on the type of data presented here); if *P* < 0.05, the difference was considered statistically significant. ROC curves were used to calculate the cutoff point, sensitivity and specificity.

### Ethics approval

All volunteers were informed that their saliva would be collected to assay the concentration of proteases and that premolar teeth extracted for orthodontic purposes would be used to isolate PDLCs; as well, identifying individual medical information was collected during or after experiments. All data, information and samples were used for our research only. A statement to the volunteers of our commitment to protect their personal information was also provided. Written informed consent for the collection of saliva, premolar teeth, and identifying individual medical information was obtained from those who participated in the experimental investigation.

The Committee for Ethics Approval of the West China School of Stomatology, Sichuan University and State Key Laboratory of Oral Diseases approved this study (IRB Reference Number: WCHS-IRB-D-2015-147).

All of the materials and methods are available.
